# Changes in Self-Efficacy and Coping Associated with Implementation of a Faculty Clinical Support Model Among Nursing Internship Students: A Single-Group Pre-Post Study

**DOI:** 10.3390/healthcare14213787

**Published:** 2026-11-05

**Authors:** Asim Alhejaili

**Affiliations:** Department of Medical-Surgical Nursing, College of Nursing, Taibah University, Al-Madinah 42353, Saudi Arabia; ahojeli@taibahu.edu.sa

**Keywords:** nursing internship, self-efficacy, coping, faculty mentorship, clinical placement, nursing education, Saudi Arabia, pre-post study

## Abstract

**Highlights:**

**What are the main findings?**

- Small but statistically significant increases in coping (d = 0.39) and self-efficacy (d = 0.21) were observed among nursing interns over a six-month internship period during which a structured Faculty Clinical Support Model was implemented.
- For both outcomes, most interns’ scores were unchanged and the mean changes were below the minimal detectable change in each scale; 85.1% of participants would recommend the model to future cohorts.

**What are the implications of the main findings?**

- Structured faculty mentorship was highly acceptable to nursing interns and warrants further evaluation as a way to support their coping resources during the transition to clinical practice.
- Given the single-group design, findings should be treated as preliminary; controlled trials are needed before the model’s causal effectiveness can be confirmed.

**Abstract:**

Background/Objectives: Nursing internship students face significant clinical stressors that may compromise wellbeing, self-efficacy, and coping. Evidence on structured faculty-led support in Middle Eastern nursing education remains limited. This study examined changes in nursing interns’ self-efficacy and coping over a six-month internship period during which a Faculty Clinical Support Model (FCSM) was implemented. Methods: A quasi-experimental, single-group pre-post design without a control group was employed. Of 172 eligible interns at Taibah University, Madinah, Saudi Arabia, 114 completed paired assessments at baseline (T0) and six months (T1). Self-efficacy was measured using an internship-contextualized Generalized Self-Efficacy Scale (GSE) and coping using the Placement Coping Scale (PCS); a five-item form assessed perceptions of the FCSM. Wilcoxon signed-rank tests on total scores were the primary analyses, with paired *t*-tests as sensitivity analyses; item-level (Holm-corrected) and gender-stratified analyses were exploratory. Results: Self-efficacy (M = 8.07 to 8.11; mean difference 0.04, 95% CI 0.005–0.08; *p* = 0.022; d = 0.21) and coping (M = 7.41 to 7.54; mean difference 0.14, 95% CI 0.07–0.20; *p* < 0.001; d = 0.39) increased slightly, but most participants’ scores were unchanged and mean changes were below each scale’s minimal detectable change. No gender × time interaction was found. The FCSM was rated favorably (M = 4.12/5.0), with 85.1% recommending it. Conclusions: Small, statistically significant increases in coping and self-efficacy followed FCSM implementation, but their educational significance is uncertain and, without a control condition, they cannot be attributed to the model. Controlled evaluations of structured faculty mentorship are warranted.

## 1. Introduction

The transition from academic nursing education to clinical practice represents one of the most challenging periods in the professional development of nursing students. During internship placements, students are expected to integrate theoretical knowledge with practical skills while navigating complex healthcare environments characterized by high patient acuity, interprofessional dynamics, and emotionally demanding situations [1,2]. Supporting students’ mental health on professional healthcare courses is a recognized challenge for academic staff [3], and this transition has been consistently associated with elevated levels of stress, diminished self-efficacy, and maladaptive coping responses that may compromise both student wellbeing and patient safety outcomes [4,5].

Self-efficacy, defined as an individual’s belief in their capacity to execute behaviors necessary to produce specific performance outcomes, plays a central role in determining how nursing students approach clinical challenges [6]. Higher levels of self-efficacy have been associated with improved clinical performance, greater persistence in the face of adversity, and enhanced psychological resilience among healthcare trainees [7,8]. Conversely, students with lower self-efficacy are more likely to experience anxiety, avoidance behaviors, and premature disengagement from clinical learning opportunities [8,9].

Coping strategies represent another critical determinant of intern wellbeing during clinical placements. Research has demonstrated that nursing students employ a range of adaptive and maladaptive coping mechanisms in response to placement-related stressors, including seeking social support, engaging in problem-solving, utilizing avoidance strategies, and employing emotional regulation techniques [9,10]. Students who develop effective coping strategies early in their internship tend to maintain higher levels of wellbeing throughout their clinical training, whereas those who experience significant stressors without adequate coping resources may experience persistent psychological distress [4].

Structured clinical support has been proposed as one way to address the wellbeing needs of nursing interns during clinical placements. In this paper, a Faculty Clinical Support Model (FCSM) refers to a structured, faculty-led form of support in which dedicated academic mentors provide scheduled and on-demand guidance, reflective discussion, clinical reasoning support, and facilitation of problem-solving strategies aligned with internship competencies, adapted from the placement wellbeing approach described by Rhodes et al. [11]. This differs from traditional preceptorship models that rely primarily on clinical staff, and from general peer or professional mentoring, because FCSMs maintain a direct academic link between the educational institution and the clinical environment; weak coordination between universities and training hospitals has been identified as a source of inadequate support for Saudi nursing interns [12,13]. Evidence on related approaches is encouraging but indirect. A meta-analysis of interventions targeting placement-related psychological distress in nursing students found moderate reductions in stress and anxiety, but most interventions were delivered before placement and focused on coping skills training rather than ongoing faculty support [14]. Mentoring by nurses or peers during clinical practice has been associated with gains in nursing students’ self-efficacy [15], whereas in a controlled evaluation of a clinical mentorship program, self-efficacy increased in both the mentored and the comparison groups and did not differ between them [16], suggesting that self-efficacy may also rise with clinical exposure alone.

Within the Saudi Arabian context, nursing education has undergone significant transformation aligned with Vision 2030 healthcare objectives, emphasizing the development of a competent, resilient nursing workforce capable of delivering high-quality patient care [17]. Taibah University’s College of Nursing has implemented a structured FCSM during internship rotations, wherein each faculty member serves as a designated academic mentor for approximately 5–10 internship students allocated across clinical training units. Faculty mentors provide accessible consultation, supportive feedback, and structured academic guidance during medical, surgical, and specialty unit rotations including critical care.

Despite this rationale, direct evidence on structured, faculty-led clinical support during the nursing internship is limited. Most available studies evaluate general mentoring, peer support, or pre-placement coping programs rather than a faculty-led support model delivered throughout the internship [14,16]. In Saudi Arabia, research on nursing interns has been dominated by qualitative and cross-sectional studies of interns’ experiences, preceptorship, and supervision [2,5,12]. Cross-sectional data also suggest that interns’ self-efficacy is higher later in the internship year irrespective of any specific intervention [18], which makes longitudinal assessment of change necessary but also complicates its interpretation. To the author’s knowledge, no study has examined changes in nursing interns’ self-efficacy and coping over a period in which a structured faculty-led support model was implemented, together with interns’ perceptions of such a model.

This study therefore aimed to examine changes in nursing interns’ self-efficacy and coping abilities over a six-month clinical internship period during which a structured Faculty Clinical Support Model was implemented at Taibah University, Madinah, Saudi Arabia. Specifically, the study objectives were (a) to assess changes in self-efficacy and coping scores from baseline to six-month follow-up; and (b) to evaluate nursing interns’ perceptions of the FCSM’s impact on their clinical preparedness, confidence, and coping capacity. Given the single-group design, the study was intended as a preliminary evaluation rather than a test of the model’s effectiveness.

## 2. Materials and Methods

### 2.1. Study Design

A quasi-experimental, single-group pre-post design without a control or comparison group was employed to examine changes in nursing interns’ self-efficacy and coping associated with implementation of the Faculty Clinical Support Model (FCSM) during clinical placement. Participants completed structured self-administered questionnaires at two time points: baseline (T0), prior to commencing their internship rotations, and at six months, at the end of the FCSM period (T1). Data collection took place from July 2025 to January 2026. This study was registered retrospectively, after data collection had been completed, on ClinicalTrials.gov (registration number: NCT07806734; date of registration: 1 September 2026; date of enrolment of the first participant: 1 July 2025). The study was not registered prospectively because the Faculty Clinical Support Model was implemented as a routine institutional support initiative for nursing internship students and was not recognized at the time of design and conduct as meeting the ICMJE/MDPI definition of a clinical trial. When this was identified during the journal review process, retrospective registration was completed immediately. Because registration took place after data collection had been completed, it does not provide the safeguards of prospective registration against selective outcome reporting; this is acknowledged as a limitation (Section 4.1). This design was selected as random assignment to a control condition was not feasible due to institutional requirements that all interns receive faculty mentorship support. Because this design lacks a counterfactual, observed changes cannot be conclusively attributed to the FCSM; maturation, accumulating clinical experience, adaptation to the workplace environment, regression to the mean, and other concurrent factors remain plausible alternative explanations [19,20] (see Section 4.1, Limitations and Future Research).

### 2.2. Setting and Participants

The study was conducted at the College of Nursing, Taibah University, Madinah, Saudi Arabia. Participants were undergraduate nursing students undertaking clinical internship placements across various clinical settings including medical, surgical, and specialty units such as critical care. Internship rotations took place across several Ministry of Health (MOH) and military hospitals, which varied in size, capacity, and service profile (from smaller facilities to hospitals with more than 500 beds). Interns rotated through several units during the study period rather than remaining in a single setting, and the hospital and unit to which each intern was allocated at each time point were not recorded in the study dataset; clinical setting could therefore not be included in the analysis. A census sampling approach was used; all 172 nursing interns eligible during the study period were invited to participate. Inclusion criteria required participants to be enrolled in the nursing internship program at Taibah University and to have commenced clinical rotations during the study period. Students who did not complete assessments at both time points were excluded from the paired analysis.

Of the 172 eligible interns invited, 133 completed the baseline questionnaire (T0; response rate 77.3%). Reasons for non-response among the remaining 39 students were not recorded, so the possibility of non-response bias cannot be assessed. Of the 133 baseline records, 124 had a matching six-month (T1) record and 9 had no follow-up response. After ten duplicated records (records sharing a student identification number with another record) were removed, retaining the first record for each identification number, 114 participants provided complete paired data (85.7% of baseline records), constituting the final analytical sample. The flow of participants is shown in Supplementary Figure S1.

### 2.3. Intervention: Faculty Clinical Support Model

The FCSM was a structured faculty-supervised mentorship model implemented throughout the first six months of the internship period. Each faculty member was assigned as a designated academic mentor for approximately 5–10 internship students allocated across clinical training units. Faculty mentors provided timely guidance, reflective discussion opportunities, clinical reasoning support, and facilitation of problem-solving strategies aligned with nursing internship competencies. During their rotations, interns were instructed to contact their assigned faculty mentors whenever they encountered workload-related stress, communication difficulties with staff, ethical dilemmas, confidence-related concerns, or clinical uncertainty in delivering patient care. When necessary, faculty mentors coordinated with training unit supervisors or referred interns to appropriate institutional support services [11]. In addition to this on-demand contact, the FCSM specified monthly scheduled meetings between each faculty mentor and their assigned interns throughout the six-month period (approximately six meetings per intern), which included reflective debriefing on stress, coping, and clinical decision-making. All participating faculty and preceptors received a brief standardized orientation covering strategies to support students’ self-efficacy (e.g., structuring graded mastery experiences, providing constructive feedback) and adaptive coping prior to the intervention period. However, beyond this specification, attendance at scheduled meetings, the actual frequency and duration of on-demand contacts, and consistency of content delivery across individual mentors were not formally logged or monitored during the study; the effective intervention “dose” received by individual participants is therefore not known with precision and is acknowledged as a limitation (Section 4.1).

### 2.4. Instruments

Generalized Self-Efficacy Scale (GSE). Self-efficacy was measured using a 10-item internship-contextualized version of the Generalized Self-Efficacy Scale developed by Schwarzer and Jerusalem [21], assessing confidence in problem-solving, goal persistence, stress management, and clinical reasoning during placement. The original GSE is a unidimensional measure whose single-factor structure and convergent validity with measures of optimism, work satisfaction, and wellbeing have been supported across many countries and languages [22]. To anchor the items to the internship, the wording of the original items was contextualized (e.g., adding “during the internship”, “on the ward”, or “during patient care”) while aiming to retain each item’s core self-efficacy statement, the number of items, and the unidimensional scoring; in addition, the original four-point response format was replaced by a 1–10 confidence rating to match the other study instruments. Because of these changes, the instrument is not identical to the original GSE and is referred to here as an adapted version. The items were administered in English, the language of instruction of the nursing program. Some rewordings went beyond adding context; for example, “If someone opposes me, I can find the means and ways to get what I want” was reworded as “If someone challenges me during the internship, I can find ways to respond effectively”. No formal content-validity assessment or pilot testing of the reworded items was undertaken. Validity evidence for the adapted version in this sample is reported in Section 3.2. Each item was rated on a scale from 1 (Not confident at all) to 10 (Completely confident). Higher mean scores indicated greater perceived self-efficacy.

Placement Coping Scale (PCS). Coping was assessed using a 9-item scale based on the work of Rhodes et al. [11], measuring perceived ability to cope with separation from support systems, assessment situations, interpersonal conflicts, patient death, difficult interactions, strategy development, help-seeking, self-awareness of distress, and emotional wellbeing maintenance. Items were rated on a scale from 1 (Not confident at all) to 10 (Very confident). The PCS was developed for a pilot evaluation of a placement wellbeing intervention for physiotherapy students, and its developers reported internal consistency evidence only; it has not undergone formal psychometric validation such as content-validity assessment, confirmatory factor analysis, or test–retest reliability testing, and no cross-cultural adaptation study has been conducted. The scale was selected because its items address placement stressors (assessment, conflict with staff, patient death, difficult interactions, and separation from usual support) that are shared by nursing and physiotherapy placements, and because it was designed to be sensitive to a placement support intervention of the kind evaluated here. As no content-validity, construct-validity, or cultural-adaptation procedures were undertaken before its application, the PCS findings are regarded as exploratory; the internal structure and convergent validity observed in the present sample (Section 3.2) provide initial, sample-specific evidence only and do not substitute for independent validation [23].

FCSM Evaluation Form. A five-item process evaluation form adapted from Rhodes et al. [11] was administered at T1 to assess perceived preparedness (Item 1), self-efficacy enhancement (Item 2), coping improvement (Item 3), accessibility of faculty support (Item 4), and overall intervention acceptability (Item 5). Items were rated on a five-point Likert scale from 1 (Strongly Disagree) to 5 (Strongly Agree).

### 2.5. Data Analysis

Data were analyzed using Python 3.12 (Python Software Foundation, Beaverton, OR, USA) with the SciPy version 1.13 (NumFOCUS, Inc, Austin, TX, USA), Pandas version 2.2 (NumFOCUS, Inc, Austin, TX, USA), pingouin (Raphael Vallat: Department of Psychology, University of California, Berkeley, CA, USA), and semopy libraries (Georgy Meshcheryakov and Anna A. Igolkina: Peter the Great St. Petersburg Polytechnic University, St. Petersburg, Russia). Total GSE and PCS scores (item means) were prespecified as the primary outcomes; item-level, gender-stratified, and process-evaluation analyses were exploratory.

- Instrument evidence: Internal consistency was assessed with Cronbach’s alpha and McDonald’s omega at both time points. Because a high alpha does not in itself establish validity [23], internal structure was examined using the Kaiser–Meyer–Olkin measure, Bartlett’s test of sphericity, parallel analysis, and a one-factor confirmatory factor analysis (maximum likelihood), with CFI, TLI, RMSEA, and SRMR interpreted against conventional criteria [24]. Convergent validity was examined through the Spearman correlation between GSE and PCS scores [9].
- Pre-post comparisons: The Wilcoxon signed-rank test was prespecified as the primary test because difference scores were bounded, discrete, and concentrated at zero (Section 3.3). The Shapiro–Wilk test was consistent with this but was not the sole basis for the choice, as normality tests are sensitive to minor deviations in samples of this size. Zero differences were excluded from ranking. Paired *t*-tests were reported as sensitivity analyses, because with more than 100 pairs they are robust to non-normality and yield mean differences comparable with the literature; results were considered robust when both tests agreed. Effect sizes were r = Z/√N for the Wilcoxon test [25] and Cohen’s d for repeated measures (dz = mean difference/SD of the differences) [26], interpreted as small (0.2), medium (0.5), or large (0.8) [27]; 95% confidence intervals were computed for mean differences. To distinguish statistical from practical significance, the numbers of participants whose scores increased, were unchanged, or decreased were reported, together with the standard error of measurement (SEM = SD at baseline × √(1 − α)) and the minimal detectable change (MDC95 = 1.96 × √2 × SEM) [28].
- Exploratory and supplementary analyses: Item-level *p*-values were adjusted within each scale using the Holm–Bonferroni procedure [29]. Gender-stratified changes were tested with Wilcoxon signed-rank tests, and differences in change between male and female participants were tested directly using a 2 (gender) × 2 (time) mixed-design ANOVA and a Mann–Whitney U test on change scores, because significance in one subgroup and not another does not indicate a subgroup difference [30]. Retained and non-retained baseline respondents were compared using Mann–Whitney U and Fisher’s exact tests. FCSM evaluation scores were compared with the neutral midpoint (3.0) using a one-sample *t*-test. Statistical significance was set at α = 0.05 (two-tailed).

### 2.6. Ethical Considerations

Ethical approval was obtained from the Research Ethics Committee of the College of Nursing, Taibah University (protocol code TUCN-REC-2025-123). The study was conducted in accordance with institutional guidelines and codes governing ethical research practices. Informed consent was obtained from all participants prior to their involvement. Student identification numbers were used only to link baseline and follow-up responses and were removed from the analysis dataset; responses were therefore de-identified to ensure participant confidentiality.

## 3. Results

### 3.1. Participant Characteristics

The final analytical sample comprised 114 nursing internship students who completed paired assessments at both time points. The mean age was 22.44 years (SD = 0.89), with the majority being female (n = 72, 63.2%). Most participants had completed their program within four years (n = 80, 70.2%), and all participants held regular academic status (n = 114, 100%). Demographic characteristics of the study participants are presented in Table 1. Baseline respondents who were not retained in the paired analysis (n = 19: 9 without a follow-up response and 10 duplicated records) did not differ significantly from the 114 retained participants in baseline GSE (M = 7.98 vs. 8.07; *p* = 0.753), baseline PCS (M = 6.75 vs. 7.41; *p* = 0.184), age (M = 22.84 vs. 22.44 years; *p* = 0.063), gender (female 78.9% vs. 63.2%; *p* = 0.205), or years to complete the program (*p* = 0.586) (Supplementary Table S1). Restricting the comparison to the nine respondents without any follow-up response gave similar results, except that they were slightly older (M = 23.00 vs. 22.44 years; *p* = 0.049). Because these comparisons had limited power and non-retained respondents had numerically lower baseline coping scores, attrition bias cannot be excluded.

### 3.2. Reliability and Validity Evidence for the Instruments

Internal consistency was high for both scales at both time points (GSE: α = 0.952 and 0.945; PCS: α = 0.949 and 0.940; ω = 0.94–0.95), although values this high may also indicate item redundancy and do not by themselves establish validity [23]. Sampling adequacy was good (KMO = 0.93 and 0.89), and parallel analysis supported a single factor for both scales, with the first factor explaining 70.7% (GSE) and 71.8% (PCS) of the variance. In one-factor confirmatory factor analyses at baseline, loadings were high (0.68–0.93) and SRMR was acceptable, but the other indices indicated marginal fit for the GSE (CFI = 0.94; RMSEA = 0.125) and inadequate fit for the PCS (CFI = 0.90; RMSEA = 0.181) [24]. GSE and PCS scores were moderately to strongly correlated (ρ = 0.58 and 0.60; *p* < 0.001), consistent with the expected relationship between self-efficacy and coping. These results partly support the unidimensional scoring of both scales but do not establish their construct validity among nursing interns.

### 3.3. Normality Assessment

The difference scores were highly concentrated at zero: 68 of 114 participants (59.6%) had identical total GSE scores and 87 of 114 (76.3%) had identical total PCS scores at both time points. The Shapiro–Wilk test confirmed departures from normality for both the GSE difference scores (W = 0.701, *p* < 0.001) and the PCS difference scores (W = 0.456, *p* < 0.001). The Wilcoxon signed-rank test was therefore retained as the primary inferential analysis, with paired *t*-tests reported as sensitivity analyses (Section 2.5).

### 3.4. Changes in Self-Efficacy

The overall GSE mean score increased from 8.07 (SD = 1.60) at baseline to 8.11 (SD = 1.50) at six months (N = 114; one out-of-range response—Item B1.9 recorded as 44 on a 1–10 scale—was identified during data verification as a data-entry error and corrected to 4 against the original record). The change was statistically significant on the Wilcoxon signed-rank test (W = 333.0, Z = 2.29, *p* = 0.022, r = 0.21) and on the paired *t*-test (t(113) = 2.23, *p* = 0.028), with a small effect size (d = 0.21) and a mean difference of 0.04 points (95% CI 0.005 to 0.08) on the 10-point scale (Table 2). Twenty-nine participants (25.4%) had higher scores at six months, 68 (59.6%) had identical scores, and 17 (14.9%) had lower scores. The mean change was far smaller than the minimal detectable change in the scale (MDC95 = 0.97 points; SEM = 0.35), which was exceeded by only one participant, indicating that the change is unlikely to be educationally meaningful. In exploratory item-level analyses, three items had unadjusted *p*-values below 0.05 (B1.1, B1.2, and B1.4), but none remained significant after Holm correction (adjusted *p* ≥ 0.080; Supplementary Table S2).

### 3.5. Changes in Coping

The overall PCS mean score increased from 7.41 (SD = 1.90) at baseline to 7.54 (SD = 1.71) at six months (N = 114). The change was statistically significant on the Wilcoxon signed-rank test (W = 0.0, Z = 4.55, *p* < 0.001, r = 0.43) and on the paired *t*-test (t(113) = 4.17, *p* < 0.001), with a small effect size (d = 0.39) and a mean difference of 0.14 points (95% CI 0.07 to 0.20) (Table 2). The Wilcoxon statistic of W = 0 reflects the fact that no participant had a lower total PCS score at six months: 27 participants (23.7%) had higher scores and 87 (76.3%) had identical scores. Because d expresses the mean change relative to the small variability of the change scores, it overstates the size of the change on the original scale; the mean change was well below the minimal detectable change in the scale (MDC95 = 1.19 points; SEM = 0.43), which was exceeded by four participants (3.5%). The change in coping should therefore be regarded as statistically detectable but of uncertain educational significance. In exploratory item-level analyses, eight of nine items had unadjusted *p*-values below 0.05 on the Wilcoxon test; after Holm correction, only coping with separation from usual support systems (C1.1; adjusted *p* = 0.043) remained significant (Supplementary Table S3).

### 3.6. FCSM Process Evaluation

Participants reported a positive evaluation of the Faculty Clinical Support Model across all five evaluation items (Table 3). The overall mean FCSM score was 4.12 (SD = 0.39) on a 5-point scale, significantly above the neutral midpoint of 3.0 (t = 30.87, *p* < 0.001). The highest-rated item was recommendation of the FCSM to future internship students (D1.5; M = 4.31, SD = 0.72), with 85.1% of participants indicating agreement or strong agreement. Accessibility of faculty support was similarly highly rated (D1.4; M = 4.26, SD = 0.76; 86.0% agreement). The item with the lowest mean, though still above the neutral midpoint, was coping improvement (D1.3; M = 3.84, SD = 0.92; 66.7% agreement).

### 3.7. Subgroup Analysis by Gender

**Figure 1:**
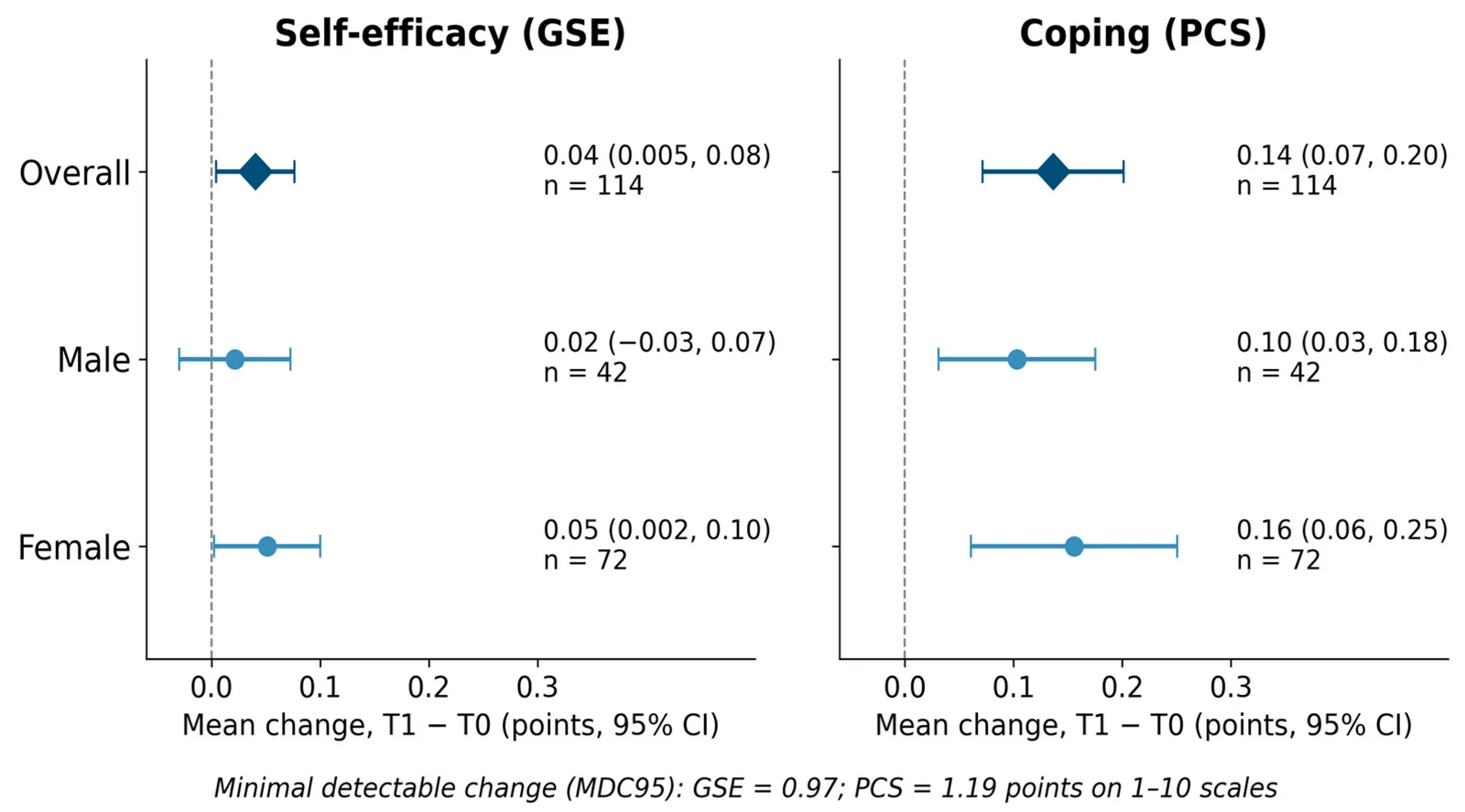
Mean Change (T1 − T0) in Total Self-Efficacy (GSE) and Coping (PCS) Scores with 95% Confidence Intervals, Overall and by Gender. The dashed line indicates no change; changes should be read against the minimal detectable change in each scale (GSE 0.97; PCS 1.19 points).

Gender-stratified results are presented in Table 2 and Figure 1. Among female participants, GSE (*p* = 0.035) and PCS (*p* < 0.001) scores increased significantly; among male participants, PCS scores increased significantly (*p* = 0.005), whereas the change in GSE scores was not significant (*p* = 0.311). However, the magnitude of change did not differ between male and female participants for either outcome (gender × time interaction for GSE: F(1, 112) = 0.64, *p* = 0.427, partial η^2^ = 0.006; Mann–Whitney U test on change scores, *p* = 0.926; for PCS: F(1, 112) = 0.60, *p* = 0.440, partial η^2^ = 0.005; Mann–Whitney *p* = 0.928). Significance within one subgroup and non-significance within another therefore does not indicate a gender difference [30], and these exploratory analyses provide no evidence that change differed by gender.

## 4. Discussion

This study examined changes in nursing interns’ self-efficacy and coping abilities over a six-month clinical internship period during which a Faculty Clinical Support Model (FCSM) was implemented at Taibah University, Saudi Arabia. Small but statistically significant increases were observed in both outcomes (self-efficacy d = 0.21; coping d = 0.39). However, most interns’ total scores did not change, the mean changes (0.04 and 0.14 points on 10-point scales) were well below the minimal detectable change in each scale, and, in the absence of a control group, the changes cannot be attributed to the FCSM. The findings should therefore be read as preliminary, hypothesis-generating evidence of change following implementation rather than as evidence of effectiveness. Process evaluation data indicated favorable participant ratings of the FCSM, supporting its perceived acceptability, although acceptability should not be equated with demonstrated effectiveness.

The very small increase in self-efficacy is consistent with the view that self-efficacy is a relatively stable construct that changes gradually in response to accumulated mastery experiences [6,22]. Bandura’s social cognitive theory posits that self-efficacy beliefs are primarily influenced through four mechanisms: mastery experiences, vicarious learning, verbal persuasion, and physiological states [6]. It is theoretically plausible that structured faculty guidance, reflective discussion, and problem-solving facilitation could strengthen self-efficacy through mastery experiences and verbal persuasion; however, these mechanisms were not measured in this study, and this interpretation is offered as a hypothesis for future testing rather than as an explanation of the findings. An equally plausible explanation is that self-efficacy increases with accumulating clinical experience during the internship, irrespective of any specific support model: Saudi nursing interns further into their internship report higher self-efficacy [18], and in a controlled evaluation of clinical mentorship, self-efficacy increased similarly in mentored and non-mentored students [16]. Regression to the mean and ceiling effects may also have contributed, given the moderately high baseline scores (M = 8.07 on a 10-point scale) [20].

The larger standardized change in coping (d = 0.39) should also be interpreted with caution. Because 76.3% of participants reported identical PCS scores at both time points and none reported lower scores, the standardized effect reflects a small, consistent upward shift in a minority of participants rather than a substantial change in the group as a whole, and the mean change of 0.14 points was well below the minimal detectable change in the scale. The exploratory item-level results showed nominal increases on most coping items, but only coping with separation from usual support systems remained significant after correction for multiple comparisons. Theoretically, a larger change in coping than in self-efficacy would be consistent with the view that self-efficacy is a relatively enduring, trait-like belief system, whereas coping is more state-like and responsive to situational resources such as knowing where to seek help [9,31]. Within Lazarus and Folkman’s transactional model of stress and coping, coping responses are shaped by an individual’s cognitive appraisal of available resources [31], and it is conceivable that the explicit provision of a named faculty contact altered interns’ appraisal of their coping resources more readily than their generalized confidence in their own capabilities. These mechanisms were not measured, however, and this account should be treated as a hypothesis to be tested—for example, by measuring appraisal of support resources and help-seeking alongside coping in a controlled design—rather than as an established explanation. Adaptation to the clinical environment over six months could equally account for the observed pattern [1].

The favorable evaluation of the FCSM (overall M = 4.12/5.0), with 85.1% of participants recommending the model to future cohorts, indicates that the model was acceptable to nursing interns. The highest-rated aspects were accessibility of faculty support and overall recommendation, suggesting that the structural features of the model, particularly the assignment of dedicated faculty mentors, were well-received by nursing interns. These findings echo the work of Rhodes et al. [11], who demonstrated that structured wellbeing support during clinical placements was positively evaluated by physiotherapy students, and extend this evidence to the nursing internship context. It is important to note that student satisfaction is a positive indicator of acceptability but is distinct from evidence of clinical effectiveness or broader implementation feasibility; the present process evaluation data primarily support acceptability, while feasibility at scale would require additional indicators such as mentor adherence rates, actual intervention dose delivered, implementation barriers, and resource requirements, none of which were formally assessed here.

In the gender-stratified analyses, female participants showed statistically significant increases in both self-efficacy and coping, whereas male participants showed a significant increase in coping only. Because the gender × time interaction was not significant for either outcome, these results do not indicate that change differed between male and female interns [30]; the non-significant self-efficacy result among male participants is also compatible with the lower statistical power of the smaller male subgroup (n = 42). Possible explanations for gender differences, such as differences in help-seeking or in engagement with mentorship [2,32,33], were not measured and are not supported by the present data; whether support needs or responses to faculty mentorship differ by gender is a question for adequately powered studies designed to test interactions.

These findings should be interpreted within the context of Saudi Arabia’s healthcare transformation under Vision 2030, which emphasizes workforce development and quality improvement in healthcare education [17]. The implementation of the FCSM at Taibah University shows that a structured faculty support model can be delivered within a Saudi nursing internship program and is well received by interns; whether such a model improves intern wellbeing and clinical readiness remains to be established in controlled evaluations.

### 4.1. Limitations and Future Research

This study has several limitations, each of which points to a specific direction for future research. First, without a control group, the observed changes cannot be attributed to the FCSM; maturation, growing clinical experience, adaptation to the workplace, regression to the mean, and concurrent events offer plausible alternative explanations [19,20]. Future evaluations should include a concurrent comparison group, for example through a stepped-wedge or cluster-randomized rollout across cohorts or sites, or at least a matched historical cohort, to separate change attributable to the model from change over time. Second, no a priori power calculation was performed. The sample was set by the census of eligible interns and was not powered to detect small effects or subgroup differences, particularly in the gender analyses. Future sample sizes should be based on the smallest educationally meaningful change, using the variability and minimal detectable change estimates reported here. Third, validity evidence for the instruments is limited. The GSE items were reworded for the internship context and rated on a 1–10 scale, and the PCS was developed in a physiotherapy pilot without formal validation. Although both scales were internally consistent and largely unidimensional, confirmatory fit was marginal for the GSE and inadequate for the PCS, and content validity was not formally assessed; the PCS findings should therefore be regarded as exploratory. Future work should assess content validity through expert review and test factor structure and measurement invariance in larger, independent samples.

Fourth, all outcomes were self-reported and open to social desirability and response-shift bias, and the large number of identical scores at both time points suggests limited sensitivity to change; observer-rated clinical performance or behavioral indicators, such as help-seeking contacts, would strengthen future evaluations. Fifth, of 172 eligible interns, 133 completed the baseline assessment and 114 provided paired data. Reasons for non-response and dropout were not recorded, and although retained and non-retained respondents did not differ significantly at baseline, this comparison had limited power, so selection and attrition bias cannot be ruled out. Future studies should record these reasons and use multiple imputation or mixed-effects models for missing data. Sixth, the single-institution sample, in which all students had regular academic status, limits generalizability. Finally, because the study was registered retrospectively, it lacks the protection against selective outcome reporting that prospective registration provides; future trials should be prospectively registered with a published protocol.

### 4.2. Theoretical and Practical Implications

Theoretically, the pattern of findings, a small detectable change in situational coping and minimal change in generalized self-efficacy, is consistent with transactional stress and coping theory [31] and with social cognitive theory’s view of self-efficacy as a belief built gradually through mastery experiences [6]. Because appraisal, mastery experiences, and help-seeking were not measured, the findings neither confirm nor refute these mechanisms but raise testable questions: whether faculty support works mainly by changing interns’ appraisal of available resources, whether changes in coping precede changes in self-efficacy, and whether the dose of support relates to the size of change. The contrast between favorable process ratings and small outcome changes also suggests that interns may value aspects of faculty support, such as reassurance, accessibility, and connection with the college, that general scales do not capture.

Practically, because the design does not establish effectiveness, recommendations should be cautious. First, faculty support models should be introduced with a planned evaluation that includes a comparison group, fidelity monitoring, and instruments validated for nursing interns. Second, mentoring can be organized explicitly, with named faculty mentors, small mentee groups, regular scheduled contacts (e.g., monthly), and coordination with unit preceptors, a gap identified by Saudi nursing interns [2,13]. Third, support could be matched to the demands of each rotation, although unit-level effects were not examined here. In critical care nursing, where interns face high acuity, sudden deterioration, and patient death, structured debriefing after such events may help. In medical nursing, heavy workloads and patients with complex multimorbidity call for a focus on prioritization and escalation of care, while in surgical nursing, rapid perioperative turnover makes communication and early recognition of postoperative complications key mentoring priorities. This fits evidence that coping-focused interventions reduce placement-related distress [14]. Fourth, mentors and preceptors need training and protected time, as supervisors’ preparation shapes the quality of internship supervision [12]. Finally, the gender-stratified results do not justify gender-differentiated mentoring; this needs adequately powered studies testing gender × time interactions.

## 5. Conclusions

Over a six-month clinical internship period during which a structured Faculty Clinical Support Model was implemented, nursing interns showed small, statistically significant increases in coping and self-efficacy. The change was larger for coping than for self-efficacy, but for both outcomes most interns’ scores were unchanged and the mean changes were below the minimal detectable change, so their educational significance is uncertain. Because the study had no control group and the instruments have limited validity evidence in this population, the changes cannot be attributed to the model. Participants rated the FCSM favorably, particularly its accessibility, supporting its acceptability. These findings provide preliminary evidence that justifies, but does not replace, a controlled, adequately powered, and fidelity-monitored evaluation of faculty-led clinical support in nursing internship programs.

## Figures and Tables

**Table 1:** Demographic and Academic Characteristics of Participants (N = 114).

Characteristic	n or Mean ± SD	%
Age (years), Mean ± SD	22.44 ± 0.89	—
Gender		
Male	42	36.8
Female	72	63.2
Years to Complete Program		
Four years or less	80	70.2
Four and a half years or more	34	29.8
Academic Status		
Regular	114	100.0

**Table 2:** Pre-Post Change in Total Self-Efficacy (GSE) and Coping (PCS) Scores, Overall and by Gender.

Outcome/Group	n	T0, M (SD)	T1, M (SD)	MD (95% CI)	*p*	r	d	↑/=/↓
Self-efficacy (GSE)								
Overall	114	8.07 (1.60)	8.11 (1.50)	0.04 (0.005, 0.08)	0.022 *	0.21	0.21	29/68/17
Male	42	8.19 (1.55)	8.21 (1.46)	0.02 (−0.03, 0.07)	0.311	0.16	0.13	12/22/8
Female	72	8.01 (1.63)	8.06 (1.53)	0.05 (0.002, 0.10)	0.035 *	0.25	0.25	17/46/9
Coping (PCS)								
Overall	114	7.41 (1.90)	7.54 (1.71)	0.14 (0.07, 0.20)	<0.001 *	0.43	0.39	27/87/0
Male	42	7.61 (1.81)	7.71 (1.72)	0.10 (0.03, 0.18)	0.005 *	0.43	0.45	10/32/0
Female	72	7.29 (1.96)	7.44 (1.71)	0.16 (0.06, 0.25)	<0.001 *	0.43	0.39	17/55/0

Note: T0 = baseline; T1 = six months; M = mean; SD = standard deviation; MD = mean difference (T1 − T0); CI = confidence interval; *p* from the Wilcoxon signed-rank test; r = Z/√N, where Z is the standardized Wilcoxon statistic; d = Cohen’s d for repeated measures (dz = mean difference/SD of differences); ↑/=/↓ = number of participants whose score increased/was unchanged/decreased. Paired *t*-tests (overall): GSE t(113) = 2.23, *p* = 0.028; PCS t(113) = 4.17, *p* < 0.001. Gender × time interaction (mixed-design ANOVA): GSE F(1, 112) = 0.64, *p* = 0.427; PCS F(1, 112) = 0.60, *p* = 0.440. Minimal detectable change (MDC95): GSE 0.97; PCS 1.19 points. One out-of-range GSE item response (44) was corrected to 4 after verification against the original record. Gender-stratified results are exploratory and not adjusted for multiple comparisons. Item-level results are given in Supplementary Tables S1 and S2. * *p* < 0.05.

**Table 3:** Faculty Clinical Support Model (FCSM) Evaluation (N = 114).

Evaluation Item	Mean	SD	% Agree
D1.1 Preparedness for challenges	3.98	0.93	74.6
D1.2 Improved confidence	4.20	0.86	79.8
D1.3 Coping with stress	3.84	0.92	66.7
D1.4 Accessibility of support	4.26	0.76	86.0
D1.5 Recommendation to others	4.31	0.72	85.1
Overall FCSM Score	4.12	0.39	—

Note: % Agree = proportion rating 4 (Agree) or 5 (Strongly Agree). Items: D1.1 “The Faculty Clinical Support Model helped me prepare for challenges encountered during clinical placement”; D1.2 “The model improved my confidence in managing clinical responsibilities”; D1.3 “The support positively influenced my ability to cope with stress during placement”; D1.4 “I knew how to access faculty support when needed”; D1.5 “I would recommend the Faculty Clinical Support Model to future internship students”. SD = standard deviation. One-sample *t*-test vs. neutral midpoint (3.0): t = 30.87, *p* < 0.001.

## Data Availability

The data presented in this study are available on request from the corresponding author due to institutional and participant privacy restrictions on student-level survey data.

## Supplementary Materials

The following supporting information can be downloaded at: https://www.mdpi.com/article/10.3390/healthcare14213787/s1, Figure S1: Flow of participants through the study; Table S1: Baseline characteristics of participants retained and not retained in the paired analysis; Table S2: Generalized Self-Efficacy Scale item-level pre-post comparison with Holm-adjusted *p*-values; Table S3: Placement Coping Scale item-level pre-post comparison with Holm-adjusted *p*-values.
