# Supplementary material for: Changes in Self-Efficacy and Coping Associated with Implementation of a Faculty Clinical Support Model Among Nursing Internship Students: A Single-Group Pre-Post Study
Source: Healthcare (Basel). 2026 Nov 5;14(21):3787. doi: 10.3390/healthcare14213787 (PMC13762505; doi:10.3390/healthcare14213787)
Supplement: Supplementary file 1 [file healthcare-14-03787-s001.zip › healthcare-4533658-supplementary.pdf]

Supplementary Materials

Changes in Self-Efficacy and Coping Associated with Implementation of a Faculty Clinical Support Model Among Nursing Internship Students: A Single-Group Pre-Post Study

Asim Alhejaili

Figure S1. Flow of participants through the study

Table S1. Baseline characteristics of participants retained and not retained in the paired analysis.

Table S2. Generalized Self-Efficacy Scale item-level pre–post comparison with Holm-adjusted p-values

Table S3. Placement Coping Scale item-level pre–post comparison with Holm-adjusted p-values

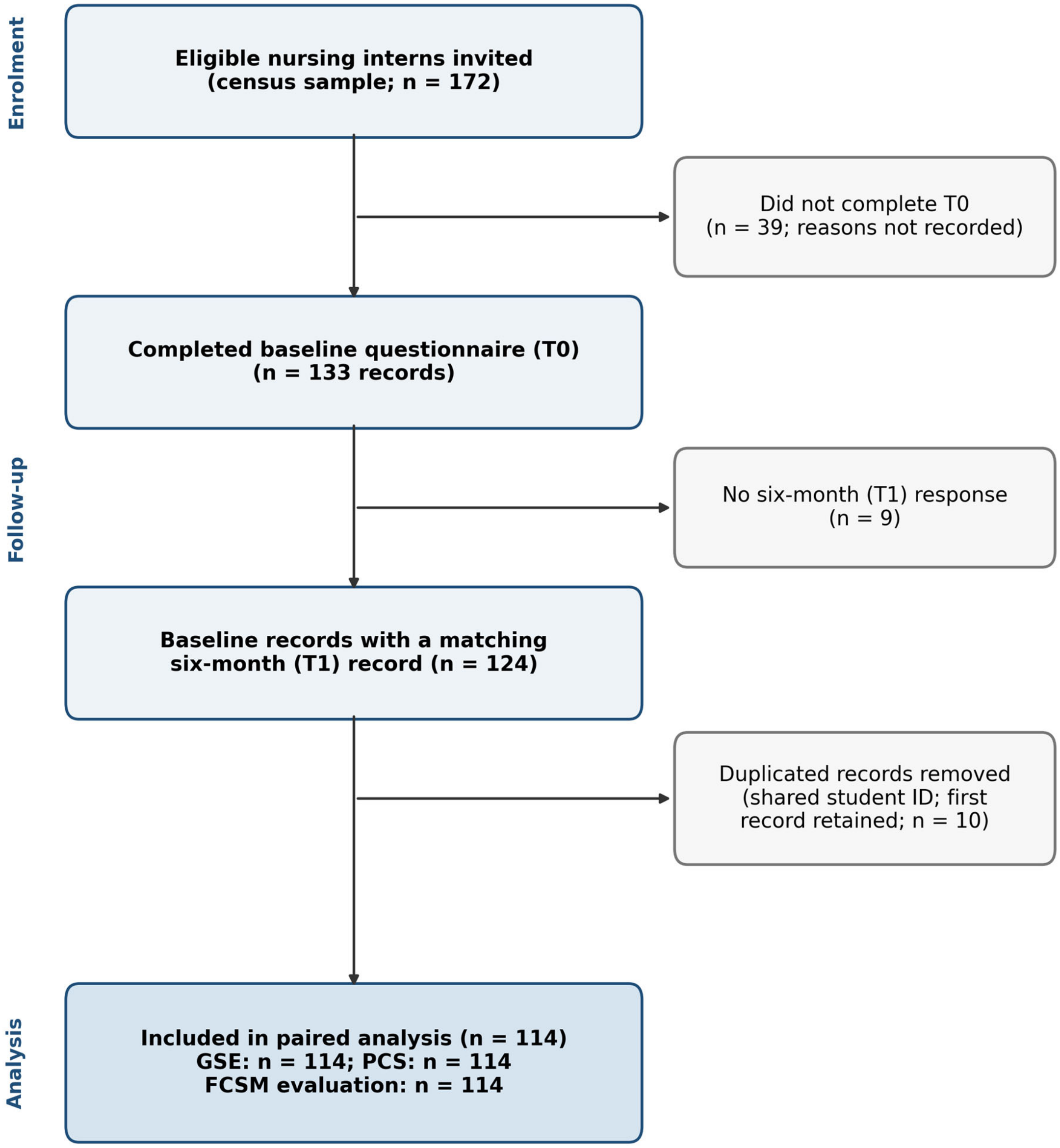

Figure S1. Flow of participants through the study.

Note: T0 = baseline; T1 = six months. Ten records shared a student identification number with another record; for each identification number, the first record was retained. Reasons for non-response and non-retention were not recorded. GSE = Generalized Self-Efficacy Scale (internship-contextualized); PCS = Placement Coping Scale; FCSM = Faculty Clinical Support Model.

Table S1. Baseline characteristics of participants retained and not retained in the paired analysis.

Characteristic	Retained (n = 114)	Not retained, all (n = 19)	*p*	No T1 response only (n = 9)	*p*
Baseline GSE score, M (SD)	8.07 (1.60)	7.98 (1.65)	0.753	8.30 (1.36)	0.838
Baseline PCS score, M (SD)	7.41 (1.90)	6.75 (1.93)	0.184	6.73 (1.67)	0.238
Age (years), M (SD)	22.44 (0.89)	22.84 (0.90)	0.063	23.00 (0.87)	0.049*
Female, n (%)	72 (63.2)	15 (78.9)	0.205	5 (55.6)	0.727
Program completed in ≤ 4 years, n (%)	80 (70.2)	15 (78.9)	0.586	6 (66.7)	1.000

Note: Retained = participants included in the paired analysis (n = 114). Not retained, all = baseline respondents excluded from the paired analysis (n = 19: 9 without a six-month response and 10 duplicated records). No T1 response only = the 9 baseline respondents without any six-month response. Continuous variables were compared with the Mann–Whitney U test and categorical variables with Fisher's exact test (expected cell counts < 5); each p-value compares the respective group with the retained participants. GSE and PCS scores are item means on 1–10 scales. *$p < 0.05$.

Table S2. Generalized Self-Efficacy Scale (GSE): item-level pre–post comparison with Holm-adjusted *p*-values (N = 114).

Item	Wording	T0, M (SD)	T1, M (SD)	MD	↑/=/↓	*p*	*p* (Holm)
B1.1	I can always solve problems (i.e., health, training, financial, social, etc.) if I try hard enough	8.18 (2.00)	8.27 (1.86)	0.10	10/103/1	0.008	0.080
B1.2	If someone challenges me during the internship, I can find ways to respond effectively.	8.32 (1.80)	8.39 (1.70)	0.08	8/105/1	0.021	0.188
B1.3	It is easy for me to stay focused on my nursing goals and complete my clinical tasks.	8.37 (1.87)	8.42 (1.78)	0.05	6/104/4	0.458	0.917
B1.4	I am confident that I can manage unexpected situations that arise during patient care.	7.58 (2.09)	7.64 (2.00)	0.06	5/109/0	0.038	0.307
B1.5	Thanks to my resourcefulness, I can handle unforeseen challenges.	7.75 (2.16)	7.68 (2.01)	−0.07	3/101/10	0.210	0.899
B1.6	I can solve most problems if I put in the necessary effort.	8.54 (1.72)	8.54 (1.61)	0.01	4/106/4	0.763	0.917
B1.7	I can stay calm when facing stressful situations because I trust my coping abilities.	8.18 (2.01)	8.22 (1.92)	0.04	4/108/2	0.236	0.899
B1.8	When I face a clinical issue, I can usually think of several possible solutions.	8.23 (1.62)	8.25 (1.59)	0.03	3/111/0	0.083	0.583
B1.9	If I face difficulties on the ward, I can usually come up with a solution.	7.75 (1.87)	7.81 (1.84)	0.06	7/104/3	0.131	0.783
B1.10	I can generally handle whatever comes my way during the internship.	7.88 (1.94)	7.92 (1.91)	0.04	2/112/0	0.180	0.899

Note: N = 114. T0 = baseline; T1 = six months; M = mean; SD = standard deviation; MD = mean difference (T1 − T0); ↑/=/↓ = number of participants whose item score increased/was unchanged/decreased; *p* = Wilcoxon signed-rank test (unadjusted); *p* (Holm) = *p*-value adjusted for 10 comparisons within the scale using the Holm–Bonferroni procedure. Items were rated from 1 (Not confident at all) to 10 (Completely/Very confident). Item-level analyses were exploratory. *Holm-adjusted $p < 0.05$. One out-of-range T1 response to Item B1.9 (recorded as 44) was corrected to 4 after verification against the original record.

Table S3. Placement Coping Scale (PCS): item-level pre–post comparison with Holm-adjusted *p*-values (N = 114).

Item	Wording	T0, M (SD)	T1, M (SD)	MD	↑/=/↓	*p*	p (Holm)
C1.1	I feel able to cope with being away from my usual support system during the internship.	7.02 (2.28)	7.25 (2.18)	0.24	10/104/0	0.005	**0.043***
C1.2	I feel able to cope with being evaluated or assessed during the internship.	7.56 (2.27)	7.67 (2.14)	0.11	6/108/0	0.026	0.159
C1.3	I feel able to cope if I experience conflict with a team member or supervisor during the internship.	7.40 (2.16)	7.50 (2.01)	0.10	4/110/0	0.066	0.159
C1.4	I feel able to cope emotionally if a patient I am caring for dies.	7.19 (2.38)	7.37 (2.16)	0.18	7/107/0	0.017	0.119
C1.5	I feel able to handle difficult interactions with patients or their families.	7.39 (1.88)	7.50 (1.79)	0.11	5/109/0	0.042	0.159
C1.6	I have developed personal strategies to deal with internship-related challenges.	7.54 (2.13)	7.62 (2.03)	0.08	5/109/0	0.039	0.159
C1.7	If I need support or advice during the internship, I know where to get help.	7.32 (2.50)	7.46 (2.31)	0.13	8/106/0	0.011	0.086
C1.8	I can recognize when I am not coping well.	7.80 (2.18)	7.96 (1.89)	0.16	6/108/0	0.027	0.159
C1.9	I feel capable of maintaining my mental and emotional wellbeing during the internship.	7.43 (2.44)	7.57 (2.17)	0.14	6/108/0	0.027	0.159

Note: N = 114. T0 = baseline; T1 = six months; M = mean; SD = standard deviation; MD = mean difference (T1 − T0); ↑/=/↓ = number of participants whose item score increased/was unchanged/decreased; *p* = Wilcoxon signed-rank test (unadjusted); p (Holm) = *p*-value adjusted for 9 comparisons within the scale using the Holm–Bonferroni procedure. Items were rated from 1 (Not confident at all) to 10 (Completely/Very confident). Item-level analyses were exploratory. *Holm-adjusted $p < 0.05$.
